# Bifunctional Opioid/Melanocortin Peptidomimetics for Use in Neuropathic Pain: Variation in the Type and Length of the Linker Connecting the Two Pharmacophores

**DOI:** 10.3390/ijms23020674

**Published:** 2022-01-08

**Authors:** Ewa Witkowska, Magda Godlewska, Jowita Osiejuk, Sandra Gątarz, Beata Wileńska, Katarzyna Kosińska, Joanna Starnowska-Sokół, Anna Piotrowska, Piotr F. J. Lipiński, Joanna Matalińska, Jolanta Dyniewicz, Paweł K. Halik, Ewa Gniazdowska, Barbara Przewlocka, Aleksandra Misicka

**Affiliations:** 1 Faculty of Chemistry, University of Warsaw, Pasteura 1, 02-093 Warsaw, Poland; magda.god@onet.pl (M.G.); jowita.osiejuk@student.uw.edu.pl (J.O.); sandra.gatarz@student.uw.edu.pl (S.G.); bwilenska@chem.uw.edu.pl (B.W.); katarzyna.kosinska2@student.uw.edu.pl (K.K.); 2 Biological and Chemical Research Centre, University of Warsaw, 101 Zwirki i Wigury St., 02-097 Warsaw, Poland; 3 Department of Pain Pharmacology, Maj Institute of Pharmacology, Polish Academy of Sciences, 12 Smetna Str., 31-343 Krakow, Poland; joanna.starnowska@gmail.com (J.S.-S.); anna.piotrowskamurzyn@gmail.com (A.P.); barbara.przewlocka@gmail.com (B.P.); 4 Department of Neuropeptides, Mossakowski Medical Research Institute Polish Academy of Sciences, Pawińskiego 5, 02-106 Warsaw, Poland; plipinski@imdik.pan.pl (P.F.J.L.); jmatalinska@imdik.pan.pl (J.M.); jdyniewicz@imdik.pan.pl (J.D.); 5 Centre of Radiochemistry and Nuclear Chemistry, Institute of Nuclear Chemistry and Technology, Dorodna 16, 03-195 Warsaw, Poland; p.halik@ichtj.waw.pl (P.K.H.); e.gniazdowska@ichtj.waw.pl (E.G.)

**Keywords:** neuropathic pain, peptidomimetics, opioids, MC4 antagonist, linkers, dual target molecules, medicinal chemistry, drug discovery, rational design

## Abstract

Based on the mechanism of neuropathic pain induction, a new type of bifunctional hybrid peptidomimetics was obtained for potential use in this type of pain. Hybrids consist of two types of pharmacophores that are connected by different types of linkers. The first pharmacophore is an opioid agonist, and the second pharmacophore is an antagonist of the pronociceptive system, i.e., an antagonist of the melanocortin-4 receptor. The results of tests in acute and neuropathic pain models of the obtained compounds have shown that the type of linker used to connect pharmacophores had an effect on antinociceptive activity. Peptidomimetics containing longer flexible linkers were very effective at low doses in the neuropathic pain model. To elucidate the effect of linker lengths, two hybrids showing very high activity and two hybrids with lower activity were further tested for affinity for opioid (mu, delta) and melanocortin-4 receptors. Their complexes with the target receptors were also studied by molecular modelling. Our results do not show a simple relationship between linker length and affinity for particular receptor types but suggest that activity in neuropathic pain is related to a proper balance of receptor affinity rather than maximum binding to any or all of the target receptors.

## 1. Introduction

Neuropathic pain results from a damage or from a disease of the nervous system [1]. This particular type of pain often becomes chronic and drugs in current medicinal use have limited effect on it. For example, the use of conventional opioid agonists for the treatment of chronic pain with neuropathic component is limited due to their weaker analgesic effects and the potential occurrence of undesirable side effects such as constipation, respiratory depression, tolerance, and dependence [2,3,4,5]. Moreover, after the first post-injury period, when the endogenous antinociceptive (opioid) system is activated, other endogenous systems come into play as counteractants of the elicited opioid activity. These systems generate pronociceptive compounds that exert their effect through non-opioid receptors and weaken the effect of analgesics [5].

For these reasons, one of the most promising strategies in the search for new analgesic drugs is to design compounds that activate the opioid system and simultaneously block the pronociceptive systems [6,7]. The molecules constructed in this way offer hope for patients suffering for many months or years from neuropathic pain for whom the currently available drugs are ineffective. Combining two or three biologically active pharmacophores to form a single molecule multiplies the possible target points and thus broadens the spectrum of action of a given drug compared to the actions of pharmacophores in separate molecules. Advantages of such a drug are better selectivity, a more favorable pharmacodynamic and pharmacokinetic profile, and reduction in the risk of interaction effects that may occur when using several different compounds [8]. Several groups developed bifunctional analgesics intended to act on two or more receptors involved in pain transmission. In terms of structure, these molecules consist of an opioid agonist (frequently a ligand of the mu opioid receptor) and an agonist that blocks a certain element of pronociceptive system, e.g., neurokinin-1 (NK-1) [9,10] cholecystokinin (CCK) [11,12], neurotensin (NT) [13], nociceptin (NOP) [14], and neuropeptide FF (NPFF) [15] receptors.

**Figure 1:**
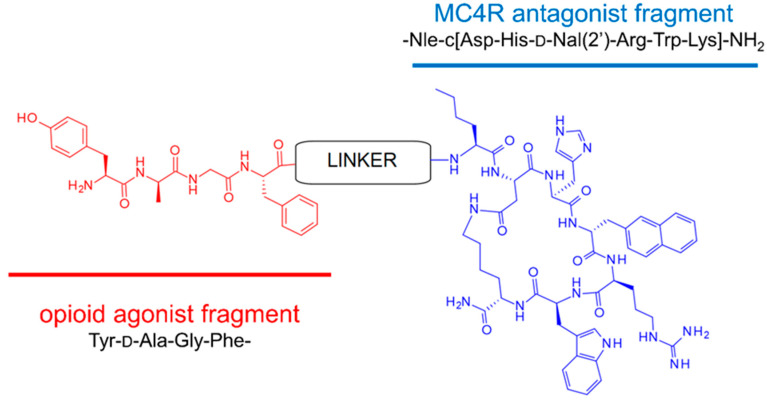
General molecular structure of bifunctional peptidomimetics **1**–**8**. In the case of compound **9**, instead of tyrosine in the first position, 2,6-dimethyltyrosine (Dmt) is used.

In our efforts along this track, based on our previous pharmacological experiments [16,17], we have designed, prepared, and evaluated for biological activity nine (**1**–**9**, general structure presented in Figure 1) bifunctional hybrid peptidomimetics [18], which consist of an opioid agonist and a melanocortin-4 (MC4) receptor antagonist. Their exact structure comprises an opioid pharmacophoric fragment based on an enkephalin analog and a melanocortin-4 receptor antagonist fragment. These elements were joined together by a set of linkers of varying length and rigidity (Table 1). Preliminary evaluation of the analgesic properties of our peptidomimetics in rodent models of acute and neuropathic pain has shown substantial differences in analgesic effects provided, depending on the linker type used to form a hybrid.

Here, we describe details on the design, synthesis, and analgesic effects of the novel compounds in animal models of acute and neuropathic pain. In addition, we present the results of opioid and melanocortin receptor affinity determinations, along with molecular modeling. We performed these to determine effect of the linker type on the analgesic activity of our compounds in neuropathic pain. Finally, we analyze data for the entire set of hybrids in search of structure–activity relationships, which may prove useful for further work.

## 2. Results

### 2.1. Design of Bifunctional Peptidomimetics

The structure of bifunctional peptidomimetics was designed in accordance with the current knowledge of the etiopathology of neuropathic pain. In this condition, a damage to the nervous system causes changes and disturbances in the proportions of the activity of endogenous anti- and pronociceptive systems. The opioid analgesic systems, which are initially activated, lose their activity over time, and the function of other neuropeptide systems becomes more intense. These changes are, inter alia, responsible for the weakening of the effect of opioid analgesics. Opioids are usually used in acute pain therapy with satisfying outcomes, but they are disappointingly ineffective in neuropathic pain. On the basis of these premises, and on the basis of our previous in-depth studies that dealt with the involvement of the melanocortin-4 receptor in pain transmission [16,17], the molecules we designed consist of two pharmacophores: an opioid agonist connected by a linker with a melanocortin-4 (MC4) receptor antagonist. The endogenous ligands of the latter have a pronociceptive effect [19].

For the opioid fragment, we used either the active tetrapeptide analog of enkephalin (Tyr-d-Ala-Gly-Phe-NH_2_), first synthesized by McGregor [20] (in compounds **1**–**8**), or a modified version thereof, Dmt-d-Ala-Gly-Phe-NH_2_ (only in compound **9**). For the MC4 antagonist fragment, we used a slightly modified SHU9119 (Ac-Nle-c[Asp-His-d-Nal(2′)-Arg-Trp-Lys]-NH_2_), one of the most effective inhibitors of the MC4 receptor, the synthesis of which had been performed originally by Hruby et al. [21]. The general molecular structure of bifunctional peptidomimetics is presented above (Figure 1).

The linkers selected to connect the pharmacophoric fragments varied in length and rigidity. All of them have an amino group and a carboxyl group that enable formation of covalent bonds to the pharmacophoric ligands. The linkers are typical amino acids (AAs) or peptides (doubled AAs), but other organic structures with rigid aromatic ring (4APhAc, 4AMB, refer to Table 1) were also used. The linkers are shortly described and classified below and in more detail in Table 1 as:

- short flexible (d-alanine, d-Ala; β-alanine, β-Ala);
- long flexible (6-aminohexanoic acid, Ahx);
- very long flexible (Ahx-Ahx);
- long rigid (4-aminophenylacetic acid, 4APhAc; 4-aminomethylbenzoic acid, 4AMB);
- long semirigid (Pro-Gly); and
- very long semirigid (Pro-Gly-Pro-Gly).

Please note that the descriptions (long/short, flexible/rigid, etc.) are relative and refer only to the set of compounds analyzed herein.

### 2.2. Synthesis of Peptidomimetics

**Figure 2:**
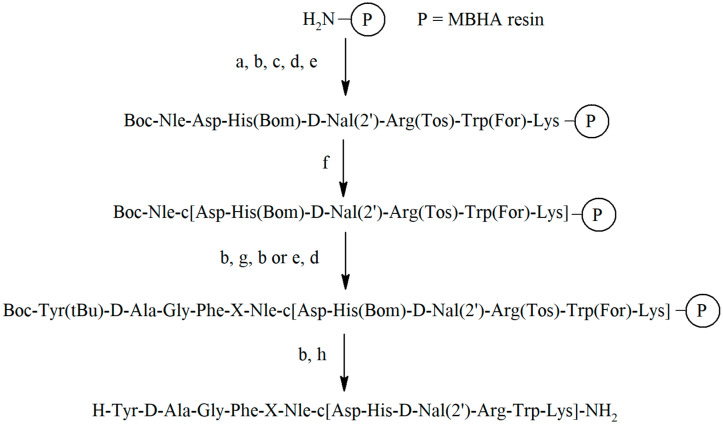
General scheme for the synthesis of the peptidomimetics **1**–**8**, in the case of compound **9** (structure shown in Table 2 instead of tyrosine in the first position, 2,6-dimethyltyrosine (Dmt) is used in the synthesis: (a) Boc-AA, DIC, HOBt, (b) trifluoroacetic acid (TFA), (c) *N*,*N*-diisopropylethylamine (DIPEA), (d) steps a–c, (e) piperidine, (f) DIC, HOBt, (g) protected linker: Boc-d-Ala or Boc-β-Ala or Fmoc-Ahx or Fmoc-4-AMB or Fmoc-4-APhAc or Boc-Gly, Boc-Pro, (h) HF.

All peptidomimetics were synthesized manually by solid phase peptide synthesis (SPPS) on MBHA resin according to the standard Boc strategy with the use of *N*,*N*′-diisopropylcarbodiimide (DIC) [24] and *N*-hydroxybenzotriazole (HOBt) [25] as coupling reagents. The removal of the peptidomimetics from the resin and elimination of the remaining protection from the functional groups of amino acids was carried out with liquid hydrogen fluoride (HF) [26]. The general scheme of the synthesis is presented in Figure 2 and Figure S1.

Crude peptidomimetics were purified using semi-preparative RP-HPLC method in reverse phase and analyzed by mass spectrometry method (ESI-MS or MALDI). Analytical data (HPLC chromatograms and MS analyses) of the obtained peptidomimetics are presented in Supplementary Materials in Table S1 and Figures S2–S10.

### 2.3. The Antinociceptive Properties

The antinociceptive properties of the peptidomimetics **1**–**9** were assessed in two types of pain models. The first one included naive mice (acute pain), and the second one included mice exposed to chronic constriction injury (CCI, neuropathic pain model). In the latter of the models, two types of stimuli (mechanical pressure and low temperature) were considered. The studied compounds were administered intraspinally, and the action of peptidomimetics was compared to the opioid (Tyr-d-Ala-Gly-Phe-NH_2_) and melanocortin-4 (SHU9119) reference compounds. In naive mice, the tail-flick test was used to evaluate the analgesic effect (acute pain model). In CCI-exposed mice, hypersensitivity to mechanical and thermal stimuli was measured by von Frey and cold-plate tests, respectively. The median effective doses (ED_50_) of the compounds tested were calculated (Table 2).

**Table 2:** Comparison of antinociceptive potency of peptidomimetics **1**–**9** and of reference compounds (enkephalin analog and SHU9119), in acute pain (tail-flick test) and neuropathic pain in CCI-exposed mice (von Frey and cold plate test). The experiments were performed on naive mice or 7–14 days after CCI procedure, and all compounds were administered intraspinally (i.t.). The results are shown as ED_50_ value with 95% confidence limits (CL) determined on the quantal data.

Code	Compound	ED50 (CL) [nmol] Naive Mice (Acute Pain) Tail-Flick	ED50 (CL) [nmol] Mice Subjected to CCI (Neuropathic Pain)	
			Von Frey (Allodynia)	Cold Plate (Hyperalgesia)
**1**	ENK-d-Ala-SHU	#	#	#
**2**	ENK-β-Ala-SHU	58.8 (27–128)	0.04 (0.03–0.68)	142 (31–654)
**3**	ENK-Ahx-SHU ^a^	#	0.0002 (0.00005–0.001)	0.004 (0.005–0.01)
**4**	ENK-(Ahx)_2_-SHU ^a^	*	0.01 (0.00002–7.8)	0.1 (0.004–3.4)
**5**	ENK-4AMB-SHU	*	0.02 (0.005–0.5)	0.16 (0.1–0.2)
**6**	ENK-4APhAc-SHU	42 (21–85)	0.02 (0.0002–1.03)	0.02 (0.0005–1.2)
**7**	ENK-Pro-Gly-SHU	#	0.4 (0.3–0.6)	0.16 (0.07–0.34)
**8**	ENK-(Pro-Gly)_2_-SHU	5 (0.4–60.3)	*	*
**9**	[Dmt^1^]ENK-Ahx-SHU ^a^	#	0.003 (0.000003–3.45)	0.009 (0.005–0.01)
	Tyr-d-Ala-Gly-Phe-NH_2_	0.05 (0.03–0.09)	0.3 (0.2–0.4)	16.3 (4.7–56)
	SHU9119 ^a^	*	3.33 (0.009–7.5)	#

*—lack of analgesic effect, # weak analgesic effects, poor dose dependency, ED_50_ cannot be calculated, ^a^ detailed in vivo study was previously presented in [27].

For peptidomimetics **4** and **5** and reference MC4 antagonist SHU9119, lack of analgesic effect in naive mice was observed. In CCI-exposed mice, any analgesic effect was not observed for peptidomimetic **8**. In a few cases, for peptidomimetic **1** (in both naive and CCI mice) and peptidomimetics **7** and **9** (in naive mice), it was impossible to calculate ED_50_ due to weak analgesic effect and poor dose dependency.

Overall, almost all synthesized peptidomimetics are more active in the neuropathic pain model and less active in the acute pain model than the opioid reference compound. These results correspond to our hypothesis of action based on changes in the activity of the pronociceptive systems (endogenous agonists of MC4 receptor among others) in neuropathic pain and not in control animals in acute pain model in which those systems are not active. The most potent peptidomimetics are compounds **3** and **9,** effective at very low doses in CCI-exposed mice, even in the thousandth or ten thousandth part of a nmol. The hybrids **4** and **6** are also quite potent. Importantly, peptidomimetics **3**, **4**, and **9** show no activity in the acute pain, while **6** exhibited some analgesic activity here. For this reason, compound **6** was not further evaluated in this study.

**Figure 3:**
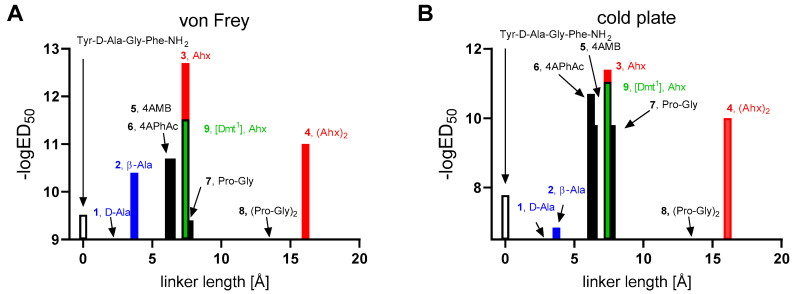
The antinociceptive effect in the neuropathic pain model in relationship to the maximal length of the linkers. The effect was measured on days 7–14 after CCI surgical procedure by the von Frey (**A**) and cold plate (**B**) tests in CCI-exposed mice (5–8 animals per group). Red marks the compounds with long flexible linkers, while blue marks the analogs with short flexible linkers. The reference enkephalin analog is marked as an empty bar. The compounds for which no bar is visible exhibited little or no antinociceptive activity. The labels show the compound code and the linker present.

Analyzing the antinociceptive results with respect to the type of the linker used (Figure 3), it is seen that among the compounds with flexible linkers, the active hybrids (**3**, **4**, and **9**) possess longer linkers (Ahx, Ahx-Ahx). On the contrary, compounds with short flexible spacers are either almost devoid of activity (**1,** d-Ala as linker) or significantly weaker (**2**, β-Ala as linker) than compound **3**.

The very active peptidomimetics **3** and **9** differ only in the amino acid in the first position. Analog **3** has tyrosine at this position, and analog **9** has 3,5-dimethyltyrosine (Dmt). We prepared analog **9** because such a substitution of amino acids usually causes an increase in antinociceptive activity of the opioid peptide analogs [28], but it was not confirmed in this case. It turned out that analogs **3** and **9** have very similar antinociceptive activity in the cold plate test, and in the case of von Frey test, analog **3** is 10 times more active that analog **9**.

Among the active compounds with rigid linkers, peptidomimetics **5** and **6** have long rigid linkers (4AMB or 4APhAc), peptidomimetic **7** possesses a semirigid long linker (Pro-Gly), while compound **8** has a very long semirigid linker.

The analogs with the aromatic linkers (4AMB, **5** or 4APhAc, **6**) have almost two orders of magnitude greater ED_50_ values than the most potent analog **3** in the von Frey test. They are, however, more potent than the analog bearing Pro-Gly dipeptidyl fragment. The analog **8** with the latter linker doubled ((Pro-Gly)_2_) exhibits very little if any activity in antagonizing allodynia and hyperalgesia.

### 2.4. Receptor Affinity

**Figure 4:**
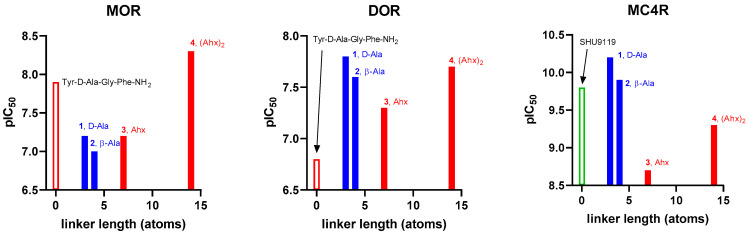
Dependence of receptor affinity (expressed as negative logarithm of IC_50_) on the linker length (n of atoms in the shortest path between the termini of the linker). The reference compound for each pharmacophore is marked as an empty bar. The blue color denotes compounds with low or absent activity in vivo, while the red color is used for the potent analogs. The labels show the compound code and the linker present.

In order to shed a light on the effect of flexible linker lengths on the peptidomimetic activity in neuropathic pain, two hybrids showing very high activity under neuropathic pain conditions (**3**, **4**) and two less active hybrids (**1**, **2**) were further tested for binding affinity to the mu and delta opioid receptors (MOR and DOR, respectively) as well as to the melanocortin-4 receptor (MC4R). The determinations for opioid receptors were performed in rat brain homogenates by competitive displacement using selective radioligands, namely [^3^H]DAMGO (a radioligand specific for MOR) and [^3^H][Ile^5,6^]DELT II (a radioligand specific for DOR). The measurement for melanocortin receptor MC4 was performed with the use of HEK293 MC4R cells and [^125^I]-NDP-α-MSH radioligand. The results are shown in Table 3 as half-maximal inhibitory concentration (IC_50_) with standard deviation (SD), and graphically in Figure 4.

Regarding the opioid receptors, the studied peptidomimetics show affinity, calculated as the IC_50_ values, between 5.47 nM (compound **4**) and 103.61 nM (compound **2**) for MOR and more consistent values between 14.46 nM (**1**) and 45.43 nM (**3**) for DOR. The opioid reference compound Tyr-d-Ala-Gly-Phe-NH_2_ binds potently to MOR (IC_50_ = 12.77 nM) and less potently to DOR (IC_50_ = 171.47 nM); these values are consistent with the previous literature reports (MOR Ki = 2.8 nM [29], DOR Ki = 300 nM [29]).

Taking into account the affinity for MOR, the tested compounds show lower affinity than the reference Tyr-d-Ala-Gly-Phe-NH_2_, with the exception of compound **4**, which belongs to the most active peptidomimetics in neuropathic pain and shows slightly higher affinity. In the case of DOR affinity, all studied compounds show higher affinity for DOR than the reference compound Tyr-d-Ala-Gly-Phe-NH_2_ does.

If we consider the length of the linkers used, the strongest DOR binder is the one with the shortest linker (**1**, IC_50_ = 14.46 nM). In the case of MOR, the opposite is true, namely: the peptidomimetic with longest linker provides the best affinity. Here, the strongest binding agent is an analog with double Ahx as linker moiety, which has an IC_50_ of 5.47 nM. This analog is also a relatively good DOR binder (IC_50_ = 22.32 nM).

In the case of the MC4 receptor, the reference compound SHU9119 exhibits subnanomolar affinity with IC_50_ = 0.15 nM. This is consistent with a recent report by Martin et al. who found for this compound Ki = 0.029 nM [30]. All our hybrid peptidomimetics display potent binding to MC4R. The weakest binder is the one with Ahx linker (**3**, IC_50_ = 1.83 nM). Further elongation of the molecule ((Ahx)_2_) yields a derivative (**4**) with subnanomolar affinity (IC_50_ = 0.50 nM), and the shorter analogs are of affinity equal to or better than the parent (IC_50_ = 0.07 nM for **1** and IC_50_ = 0.12 nM for **2**).

With respect to selectivity (calculated as ratio of IC_50_ values), compounds **1** and **2** (with shorter flexible linkers) seem to prefer binding to DOR rather than MOR. They also have a strong preference for MC4R over both opioid receptors (MC4R/MOR selectivity close to 1000, while MC4R/DOR selectivity close to 200).

On the contrary, the hybrids with longer flexible linkers exhibit much more balanced binding. For compound **3**, IC_50_ values measured for MOR and DOR are similar (ratio 0.7), while the selectivity factors MC4/MOR and MC4/DOR are only 35 and 25, respectively. The hybrid **4** is slightly selective towards MOR compared to DOR (ratio 4.1). For this compound, MC4R/MOR and MC4R/DOR selectivity are 11 and 45, respectively.

We also studied experimentally the lipophilicity of the compounds **1**–**4**. The obtained logP values vary only moderately (in the range −2.30 to −2.92, Table S2) and cannot account for the large differences in the antinociceptive activity. It seems then that the lipophilicity is not a factor responsible for the in vivo activity differences.

### 2.5. Molecular Modelling

**Figure 5:**
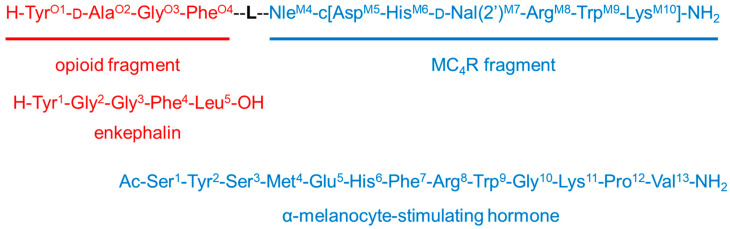
The residue numbering convention used in the description of the modelling results. The numbering is associated with the numbering of the corresponding residues in the enkephalin (O) and α-melanocyte-stimulating hormone (M) peptides. L stands for the linker.

For obtaining tentative insight into the possible structural basis of the observed affinities, the compounds **1**–**4** were modelled in the binding sites of MOR, DOR, and MC4R. In describing the results of modelling, the residue numbering convention explained in Figure 5 is used.

**Figure 6:**
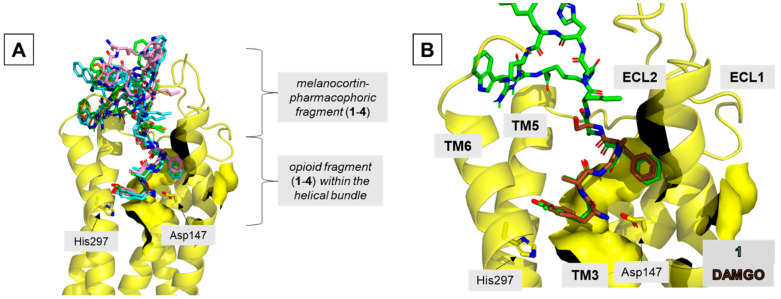
Binding poses of the studied compounds in the MOR binding site. (**A**) A general view. Superposition of compounds **1**–**4**. (**B**) Focus on the opioid fragment. Superposition of compound **1** (green) and DAMGO (brown; position taken from 6DDF structure [31]). The receptor (yellow) is shown in a simplified manner as helices (without TM1 and TM7), surface, and selected side chains shown as sticks. Ligands are shown as sticks.

In the MOR binding site, all analogs locate the enkephalin fragment in a manner similar to that experimentally found for DAMGO [31] (Figure 6 and Figure S11, Table S3). The main features of this binding mode are: (1) the ionic interaction of the protonated Tyr^O1^ amino group with Asp147^3.32^, (2) the positioning of the Tyr^O1^ phenol group close to His297^6.52^, and (3) the location of the Phe^O4^ aromatic ring in a hydrophobic subsite made of side chains belonging to transmembrane helix 3 (TM3) and extracellular loops 1 and 2 (ECL1 and ECL2).

The melanocortin-pharmacophoric fragment of **1**–**4** is located at the extracellular outlet of the binding site, above the opioid portion of the ligands (Figure 6A). In **1** and **2**, this fragment of the molecule is wedged between ECL2 and ECL3 of the receptor (Figure S12). In analog **3**, one of the top solutions is located in the same manner as in the case of **1** and **2**, while in another one, the melanocortin-pharmacophoric-fragment goes closer to closer to ECL3 and to the extracellular tip of TM1 (Figure S12). Such positioning is also found for the top-scored cluster found for **4** (Figure S12). Detailed description of the contacts formed by the melanocortin-pharmacophoric fragment of **1**–**4** with the MOR is given in Supplementary Materials (below Figure S12)

Regarding the DOR binding site, the opioid part of all hybrids (Figure S13) is predicted to have the canonical interaction between the protonated amino group and D128^3.32^. The tyrosine aromatic ring approaches Met132^3.36^, Trp274^6.48^, Ile277^6.51^, and His278^6.52^ forming hydrophobic interactions with the side chains of these residues. The locations of Phe^O4^ ring does slightly differ among the analogues (Figure S13), but in all cases, the aromatic ring is wedged between the side chains of Lys108^2.63^, Val197^ECL2^, and Arg291^ECL3^.

The melanocortin-pharmacophoric fragment of **1**–**4**, as in the case of MOR, locates at the extracellular outlet of DOR binding site, above the opioid fragment (Figure S14). In the case of DOR, however, for compounds **1**–**3** (Figure S14A–F), the MC4R-related fragment is positioned more towards TM1 and ECL1. For **1** (Figure S14A,B), docking predicts that the binding pose is stabilized by interactions of Arg^M8^ with Asp193^ECL2^ (H-bonds), lactam bond’s hydrogen with Ser45^1.28^ (H-bond) and His^M6^ with Arg41^1.24^ (hydrophobic), and Nle^M4^ carbonyl oxygen with Tyr109^2.64^ (H-bond). In the case of **2** (Figure S14C,D), the Nle^M4^ carbonyl oxygen H-bonds to Ser45^1.28^ side chain, while Trp^M9^ aromatic ring contacts Thr113^ECL1^ and Met111^2.66^ side chains. For **3** (Figure S14E,F), docking predicts the interactions of Nle^M4^ carbonyl oxygen with Ser45^1.28^ side chain (H-bond) and of Trp^M9^ side chain with Met111^2.66^ side chain (hydrophobic). For the longest derivative **4** (Figure S14G,H), Arg^M8^ is predicted to interact with Asp193^ECL2^.

At the MC4R, the melanocortin-pharmacophoric portion of the hybrids is predicted to retain the experimental position and intramolecular contacts of SHU9119, as found in the 6W25 crystal [32]. In brief, the cyclic fragment resides in the intrahelical bundle (Figure S15 and Table S5), interacting with the calcium cation by carbonyl oxygens, and forming many interactions with the receptor. These include several electrostatic interactions (between Asp126^3.29^ and Arg^M8^, Thr101^2.61^ and His^M6^, Asn123^3.26^ and Arg^M8^, Ser188^ECL2^ and Arg^M8^, Ser188^ECL2^ and Trp^M9^, and His264^6.54^ and Trp^M9^), two π–π interactions (Phe51^1.39^ to His^M6^ and Tyr268^6.58^ to Trp^M9^), and extensive hydrophobic contacts with all the transmembrane helices.

Regarding the opioid-linker fragment, its positioning differs among the analogues (Figure S16). For compound **1** (Figure S16A), it is located close to ECL1 and stabilized by a set of H-bonds between the amide hydrogens and Asp111^ECL1^ (side chain and carbonyl oxygen) and Thr112^ECL1^ (carbonyl oxygen). In analogue **2** (Figure S16B), the opioid fragment is localized between ECL1 and ECL2, being stabilized by the interaction of Tyr^O1^ amino group and Asp189^ECL2^, and of β-Ala amide hydrogen and Asp111^ECL1^. In the case of hybrid **3** (Figure S16C,E), three different positionings of the opioid fragment were found (within 1.0 kcal/mol from the lowest binding energy), with the opioid-linker fragment closer to either ECL1 or ECL2 (contacts listed in Supplementary Materials under Table S5). For the longest analogue **4** (Figure S16F), docking found a position in which the opioid-liker fragment is stabilized by H-bonds between amide hydrogens of the linker fragment and Asp111^ECL1^ and Asp189^ECL2^.

## 3. Discussion

Safe and effective treatment of neuropathic pain remains an unmet medical need. A rational strategy for developing novel promising drugs against this condition is to create hybrid analgesics. Such compounds are intended to activate the opioid receptors and simultaneously to inhibit the pronociceptive compensatory response mediated by other receptors. Working in this paradigm, we conceived the idea of bifunctional compounds containing the pharmacophores of opioid agonist and melanocortin-4 antagonist. The choice of this pair of target receptors is justified by the mutual entanglement of the opioid and melanocortin systems in the pain transmission [17,33,34]. Earlier, a related concept was tentatively examined by Lee et al. who designed bi- and trifunctional ligands based on opioid, melanocortin-4 (agonist and antagonist), and/or cholecystokinin receptor pharmacophores [35,36]. Those compounds, however, were not advanced to the in vivo studies.

In our efforts along this track, we designed and synthesized nine hybrid compounds [18,37]. Their evaluation as to the analgesic properties in rodent models of acute and neuropathic pain revealed substantial differences in the analgesic effects, depending on the linker type that was used to form a hybrid. Now, we report in detail their synthesis, additional in vivo data, receptor affinity, structure–activity relationships, and molecular modelling.

As in every basic science research paradigm that uses an experimental model to infer about the action of a novel drug, the present study has some limitations. The fact that research is carried out on a relatively small number of new hybrids is certainly one of them. Moreover, the selected neuropathic pain model is highly invasive, which limits the possibility of using more animals. Still, we hope that the currently obtained data will allow us to better approach the next stage in research on new bifunctional compounds.

Looking at the whole set of hybrids, it is to be noticed that the most potent compounds are those having long or very long flexible linkers (Ahx, **3**; (Ahx)_2_, **4**) connecting the opioid and melanocortin-4 pharmacophores. On the contrary, fairly flexible but shorter linkers (d-Ala, **1**; β-Ala, **2**) are present in less active hybrids.

For getting further understanding of these differences, we investigated four hybrids (**1**–**4**) as to their affinity for MOR, DOR, and MC4R. Surprisingly, peptidomimetic **3** (ENK-Ahx-SHU), which proved to be the most efficient in attenuating neuropathic pain-like behavioral symptoms, is not the most potent binder at any of the studied receptors. In fact, it displays the lowest affinity at both DOR and MC4R among the four compounds for which the affinities were measured. The longer analog **4** with (Ahx)_2_ linker, which is a single-digit nanomolar binder at MOR, displays much worse ED_50_ values in both von Frey and cold plate tests.

Comparing, however, the calculated selectivity between melanocortin-4 and opioid receptors, there is a visible difference between compounds showing high antinociceptive activity and those with lower potency. The less active hybrids (**1**,**2**) are significantly more MC4R-selective. On the other hand, the highly active compounds (**3**,**4**) exhibit more balanced affinity profile towards both opioid and melanocortin receptors.

In light of this, it would be interesting to have a structure-based intuition on how the linkers might affect the interactions with the singular receptors. This could enable the design of hybrids with balanced affinities using the appropriate linkers. In a tentative attempt to gain some insight, we performed basic modelling of compounds **1**–**4** in the binding sites of the target receptors. According to modeling, in the considered complexes, the substructure pharmacophoric for a given receptor resides deeper in the orthosteric pocket, while the portion responsible for binding to another receptor is located closely to the extracellular outlet of the receptor. Neither linkers nor the additional pharmacophores pose a significant obstacle to accommodating a good binding pose for the studied ligands deep in the binding site. Thus, the changes of binding affinity (in comparison to the reference compounds) are likely associated with subtle interplay of the following factors. Some additional contacts are formed by the extracellular outlet of the binding site. They are, however, weakened by high solvent exposure. Their formation results also in conformational entropic penalty (upon freezing the flexible linkers). The net effects are some significant but not dramatic changes in receptor affinities compared to the reference compounds, which are hard to predict based on the modelling. Please note that the conducted modelling has major limitations. First, the receptor side-chains close to the extracellular outlet of the binding site are likely to be relatively mobile, undergoing some conformational changes. Second, the hybrids **1**–**4** are quite large compounds with many rotatable bonds. In such cases, it is hard to ensure exhaustive sampling of the docking solutions and so the results of docking must be taken with caution. They do, however, represent some working hypotheses for future work.

Overall, in the studied set, the activity in neuropathic pain clearly depends on the type of linker used. Only peptidomimetics containing longer and flexible linkers were found to be very effective at low doses in reducing allodynia and hyperalgesia (von Frey and Cold Plate, respectively) that characterize neuropathic pain, while showing little activity in acute pain. Our results provide some tentative hints that the activity in neuropathic pain is associated with proper balance of the receptor affinities rather than with maximal binding at any or all of the target receptors. Last but not least, three of the studied hybrids demonstrate a very strong effect in CCI-exposed mice. Therefore, the structure and possible applications of the hybrids has been claimed in the Patent US11041010 [37] and Polish and international pending patents (PL422093, PCT/IB2018/054925). It can be hoped that these compounds may enable the development of a good drug effective in neuropathic pain. The best of our peptidomimetics will soon be tested for side effects (e.g., constipation, addiction). We believe that by carrying out this difficult research, even to a limited extent, we are trying to develop principles for guiding the design of a bifunctional compound for use in neuropathic pain.

## 4. Materials and Methods

### 4.1. Materials

Unless otherwise specified, reagents were obtained from commercial sources and used without further purification. MBHA resin was obtained from Bachem (Torrance, Torrance, CA, USA ), Rink Amide from Activotec (Cambridge, UK), protected amino acids and coupling reagents and the following linkers, Fmoc-4AMB-OH and Fmoc-4APhAc-OH, were purchased from Iris Biotech (Marktredwitz, Germany), and Boc-Dmt was purchased from PE Biosciences Ltd. (Hong Kong, China).

[^3^H]DAMGO (a radioligand specific for MOR) was bought from Perkin Elmer Inc., Poland, [^3^H][Ile^5,6^]DELT II (a radioligand specific for DOR) was purchased from Isotope Laboratory, Biological Research Centre, Institute of Biochemistry (Szeged, Hungary). NDP-α-MSH ([Nle^4^,d-Phe^7^]-α-MSH trifluoroacetate salt) and SHU9119 (acetyl-[Nle^4^,Asp^5^,d-2-Nal^7^,Lys^10^]-cyclo-α-MSH (4-10) amide trifluoroacetate salt) were purchased from Bachem AG, Bubendorf, Switzerland. [^125^I]-NDP-α-MSH radioligand (Product Number: NEX352; Ac-Ser-[^125^I]Tyr-Ser-Nle-Glu-His-d-Phe-Arg-Trp-Gly-Lys-Pro-Val-NH_2_) was purchased from PerkinElmer, Boston, MA, USA. HEK293 MC4R cells, which stably express the melanocortin 4 receptors (MC4R), were obtained from Innoprot (Derio, Vizcaya, Spain).

### 4.2. Synthesis and Purification of Peptidomimetics

#### 4.2.1. Synthesis of Peptidomimetics

The peptidomimetics **1**–**9** were synthesized on a MBHA resin (0.27 mmol/g), according to the standard Boc strategy. Amino acids derivatives were used in the synthesis: Boc-l-Lys(Fmoc)-OH, Boc-l-Trp(For)-OH, Boc-l-Arg(Tos)-OH, Boc-d-Nal(2′)-OH, Boc-l-His(Bom)-OH, Boc-l-Asp(OFm)-OH, Boc-l-Nle-OH, Boc-l-Phe-OH, Boc-Gly-OH, Boc-d-Ala-OH, Boc-l-Tyr(tBu)-OH, or Boc-l-Dmt-OH. The following derivatives were used to prepare the linker: Boc-d-Ala-OH (**1**), Boc-β-Ala-OH (**2**), Fmoc-*ε*Ahx-OH (**3**, **4**, **9**), Fmoc-4AMB-OH (**5**), Fmoc-4APhAc-OH (**6**), Boc-Gly-OH (**7**, **8**), and Boc Pro-OH (**7**, **8**). To remove Boc protection, a 50% solution of trifluoroacetic acid in DCM was used, while a 30% solution of piperidine in DMF was used to remove Fmoc protection. The subsequent amino acid residue was attached by a carbodiimide method with use of *N*,*N*′-diisopropylcarbodiimide (DIC) [24] as a coupling reagent in the presence of *N*-hydroxybenzotriazole (HOBt) [25] with a 3-fold molar excess of the reagents. The reaction was carried out for 2 h at room temperature. Attachment efficiency was monitored by the ninhydrin (Kaiser) test for the presence of free amine groups. If free amino groups were detected after coupling, the coupling reaction was repeated with use of HBTU or TBTU as the coupling reagent in the presence of HOBt and DIPEA. The lactam ring was formed after Boc-Nle-OH attachment to the peptide chain. For this purpose, protecting groups from pendant groups of aspartic acid (OFm) and lysine (Fmoc) residues were removed by use of a 30% solution of piperidine in DMF, followed by cyclization by the carbodiimide method for 4 h. The cyclization was repeated until the negative result of the ninhydrin test (the absence of free amine groups) was obtained. In the final step of the cyclization (at 4th or 5th repeat), the uronium salt method was applied. The cyclization reaction lasted usually 16–20 h. When the cyclization was completed, the peptide synthesis was continued until the final sequence on the resin was achieved. After removal of the Boc group from the N-terminal amino acid, the simultaneous cleavage of the peptide from the resin and of the side chain protecting groups was carried out with liquid hydrogen fluoride in the presence of anisole at 0 °C for 1 h.

#### 4.2.2. Analysis and Purification of Peptidomimetics

Analysis and purification of the obtained peptidomimetics was conducted by RP HPLC on the KNAUER liquid chromatograph. Mass spectra were registered on the Shimadzu LC–MS mass spectrometer provided with an ESI ion source (electrospray), and ion trap and time-of-flight analyzer analyses were carried out in the positive ion mode. For two compounds, MALDI Shimadzu Biotech Axima Performance with time-of-flight analyzer and cinnamic acid as the matrix was used. The analytical data of the peptidomimetics are presented in Table S1 and Figures S2–S10.

### 4.3. Behavioural Tests

#### 4.3.1. Acute Pain Model

##### Naive Mice—Tail-Flick Test

The tail-flick test was performed with use of Tail-Flick Analgesic Meter (Ugo Basile, Comerio, Italy) to evaluate the pain threshold to a thermal stimulus. During the procedure, an animal was placed on the apparatus surface and gently held by the experimenter. A beam of light was focused on a dorsal tail surface, approximately at 1 cm from the tail tip. When the animal flicked its tail, the timer stopped, and the recorded time (latency) was measured. The cut-off time to protect tissue damage was 9 s [9,27].

#### 4.3.2. Neuropathic Pain Model

To test the analgesic potential of the synthesized compounds, the model of the chronic constriction injury to the sciatic nerve (CCI), which has been used for many years in our laboratory and widely used by many other groups, was used. The surgical procedure was performed according to Bennett and Xie [38]. Under isoflurane anesthesia, an incision was made below the mouse’s right hipbone, and the sciatic nerve was exposed. Three ligatures with 4/0 silk thread were made around the nerve distal to the sciatic notch with 1 mm spacing, until a brief twitch in the respective hind limb was observed. After 7 days of recovery, mice were tested to assess the development of neuropathic pain behavior. All CCI mice developed allodynia and hyperalgesia. Main experiments were conducted on days 7–14 after CCI surgical procedure.

##### Mice Subjected to CCI—Von Frey Test

Mechanical sensitivity to non-noxious stimuli was measured by applying a set of calibrated nylon monofilaments in increasing value (0.6–6 g; Stoelting, Wood Dale, IL, USA) on a tested hind paw plantar surface, until a behavioral response was observed. Response considered as pain behavior included rapid paw withdrawal, shaking, and licking. In the von Frey test, results are expressed as pressure (g) applied with a calibrated plastic filament to the midplantar surface of the mouse’s injured hind paw (cut-off: 6 g), which elicited a foot withdrawal response [9,27].

##### Mice Subjected to CCI—Cold Plate Test

Sensitivity to noxious thermal stimuli was assessed with usage of Cold/Hot Plate Analgesia Meter, (Ugo Basile, Comerio, Italy). The temperature of the plate was kept at 2 °C, and the cut-off latency was 30 s. The mice were placed on the cold plate, and the time until the hind paw was lifted was recorded, which was considered as a reaction to noxious cold stimulus [9,27].

#### 4.3.3. Statistical Analysis

The behavioral data are presented as the percentage of the maximal possible antinociceptive effect (% MPE ± SEM), which was calculated according to the following equation: % MPE = [(TL − BL)/(CUT-OFF − BL)] × 100%, where BL is the baseline latency, and TL is the latency obtained after drug injection. The results of the experiments were statistically evaluated using one-way analysis of variance (ANOVA). The differences between the treatment groups throughout the study were further analyzed with Bonferroni post hoc tests. The Litchefield and Wilcoxon method was used to calculate the ED_50_ value (a dose necessary to produce a 50% response) with 95% confidence limits on quantal data [39].

### 4.4. Radioligand Competition Binding Assay for MOR, DOR, MC4R

#### 4.4.1. Binding Affinity Determinations for MOR and DOR

The binding affinity of peptidomimetics **1**–**9** and reference compoundsTyr-d-Ala-Gly-Phe-NH_2_ for MOR and DOR was determined in competitive radioligand binding assays according to the method previously described [40,41,42]. The specific radioligands were [^3^H]DAMGO (DAMGO (a specific MOR ligand) and [^3^H][Ile^5,6^]DELT II]) (a specific DOR ligand). Membrane fractions of rat brain homogenate were incubated at 25 °C for 60 min in the presence of radioligands (0.5 nM) specific for each receptor and the increasing concentrations of the tested compounds. For measuring non-specific binding, 10 μM naloxone was used as the competitor for opioid receptors. The reactions were carried out in assay buffer containing Tris-HCl (pH 7.4) with an addition of bovine serum albumin (BSA) and protease inhibitors (bacitracin, bestatin, captopril). After the incubation, the binding reactions were terminated by rapid filtration with M-24 Cell Harvester (Brandel/USA) through GF/B Whatman glass fiber strips (pre-soaked with 0.5% PEI in order to minimize non-specific binding). Radioactivity retained on the filters was measured in MicroBeta LS, Trilux scintillation counter (PerkinElmer, USA). The experiments were repeated at least three times in duplicate. The IC_50_ value for each compound was determined using GraphPad Prism [43].

#### 4.4.2. Binding Affinity Determinations for MC4R

##### Cell Culture

HEK293 MC4R were cultured in Earle’s Minimum Essential Medium (EMEM) supplemented with 10% heat inactivated fetal bovine serum (FBS). Cultures were grown under the humidified atmosphere of 5% CO_2_ in the air at 37 °C, up to 80% settlement of the bottle surface.

##### Radioligand Competitive Binding Assay

HEK293 MC4R cells were seeded around 10^5^ cell/well in a 24-well plates two days before the experiment. Just before experiment, cells were washed twice with 0.5 mL of binding buffer (EMEM supplemented with 25 mM HEPES buffer, 0.2% bovine serum albumin, 1 mM 1,10-phenanthroline, 0.5 mg/L leupeptin and 200 mg/L bacitracin) [44]. To determine EC_50_ value of analyzed ligand, different concentrations of non-radioactive ligand (from 0.07 pM to 10 µM) in 0.5 mL of binding buffer with fixed dose 0.15 µCi of [^125^I]-NDP-α-MSH radioligand were added to one of each well with cells and incubated for 60 min at 37 °C. The competition between ligands was ended by gentle withdrawal of medium solution, followed by cell washing with 2 × 0.5 mL of cold binding buffer, while the attached cells were lysed using 2 × 0.5 mL of 1 M NaOH. The radioactivity of collected solutions were measured on WIZARD^2^ 2480 Automatic Gamma Counter (PerkinElmer, Boston, MA, USA) in measurements with decay correlation protocol. All binding parameters were determined using GraphPad Prism Software (GraphPad Software Inc., San Diego, CA, USA). The competition assay determination was based on the normalized dose response in function of logarithm of inhibitor concentration (with variable slope) equation. All the EC_50_ values were calculated as an average of two separate competition assays performed in duplicates. In the calculation of **1**, one data point was removed due to being significant outlier.

### 4.5. Molecular Modelling

The studied hybrids were modelled in the binding sites of MOR, DOR, and MC4R using local search procedure in AutoDock 4.2.6 (Scripps Research Institute, La Jolla, California) [45].

For MOR and MC4R, the starting point for the calculations was selected by modifying and extending the crystallographic ligands (SHU9119 in the MC4R structure, 6W25 [32], DAMGO in MOR structure, 6DDF [31]—these structures were chosen for having a crystallographic ligand being a substructure of our hybrids) by attaching the linker and either the opioid or MC4R fragment. For DOR, first, an *N*-methylated (at the C-terminus) enkephalin-amide was docked to the receptor (PDB accession code: 6PT2 [46]), and a selected binding pose was used for extending the molecule with the linker and the MC4R-fragment.

The complexes prepared in the way described above were optimized using local search docking with AutoDock 4.2.6 [45] with the following parameters: 300 individuals in a population, 500 iterations of the Solis-Wets local search, local search space set to at least 10.0, and at least 1000 local search runs (2000 runs for longer analogues). The procedure was repeated at least 20 times for each compound, and for the longest analogs, it was repeated up to 160 times. If there were more than 32 rotatable bonds, the docking was repeated with different sets of the bonds allowed to rotate. The results were sequentially clustered according to the binding poses geometry, pooled, and again clustered and pooled, etc. Top scored solutions for each compound (clusters within 1.0 kcal/mol threshold from the best scored cluster) were subject to further analysis.

In the cases of docking to opioid receptors, a pharmacophoric filter was applied, according to which the solutions lacking the canonical ionic interaction between Asp^3.32^ and the protonated amine group of the peptide were discarded.

The receptor structures used for docking (MC4R: 6W25 [32], MOR: 6DDF [31] and DOR: 6PT2 [46]) were the refined models as provided by the GPCRdb service [47]. These models have the mutated residues replaced with the native ones and the side chains missing in the original PDB structures supplemented. The receptor structures were prepared for docking in AutoDockTools [45]. Of particular note is that the Ca^2+^ cation that mediates SHU9119 binding to MC4R [32] was retained as found in the crystal structure. The box was set around the experimental positions of the crystallographic ligands and extended so that to enable the docking of the studied hybrids (box sizes: 45 Å × 45 Å × 50 Å, 47 Å × 47 A × 47 Å, 40 Å × 40 Å × 50 Å for MOR, DOR and MC4R, respectively). The grids were calculated with AutoGrid 4. Molecular graphics were prepared in the Open-Source PyMOL [48].

## 5. Patents

The structure of the tested peptidomimetics was claimed in the United States patent nr US11041010 (Hybrid peptidomimetics for use in neuropathic pain, Misicka-Kęsik Aleksandra, Witkowska Ewa, Wileńska Beata, Przewłocka Barbara, Mika Joanna, Starnowska-Sokół Joanna, Piotrowska-Murzyn Anna). There are also pending Polish (PL422093) and international patents (PCT/IB2018/054925).

## Figures and Tables

**Table 1:** The linkers used in the study and the descriptors of their length and flexibility.

Linker	Length		Flexibility			Comment
	n of Atoms ^a^	Maximal Length (Distance) ^b^ [Å]	Rotatable Bonds ^c^	Rotatable Bonds Fraction ^d^	Φ Index ^e^	
d-Ala -HN-CH(CH_3_)-CO-	3	2.4	2	0.11	3.06	short flexible
β-Ala -HN-(CH_2_)_2_-CO-	4	3.7	3	0.17	3.84	short flexible
Ahx -HN-(CH_2_)_5_-CO-	7	7.4	6	0.22	6.53	long flexible
(Ahx)_2_ -(HN-(CH_2_)_5_-CO-)_2_-	14	16.1	12	0.26	12.21	very long flexible
4AMB 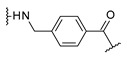	7	6.4	3	0.12	3.51	long rigid with aromatic ring
4APhAc 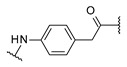	7	6.2	3	0.12	3.51	long rigid with aromatic ring
-Pro-Gly-	6	7.7	3	0.10	4.09	long semirigid (with proline)
-(Pro-Gly)_2_-	12	13.8	6	0.12	7.26	very long semirigid (with proline)

^a^ number of atoms (in the shortest path between the termini of the linker), ^b^ measured between the terminal nitrogen and carbon atoms, in the fully extended conformation, ^c^ number of rotatable bonds, the φ torsion of proline residues and the amide bonds excluded, ^d^ fraction of the number of rotatable bonds to the number of all bonds, ^e^ Φ Kier flexibility index [22] calculated on Acetyl-linker-amide model systems using the program Dragon [23].

**Table 3:** Binding affinity for the opioid receptors MOR, DOR, and melanocortin receptor MC4 of studied peptidomimetics and their reference compounds (Tyr-d-Ala-Gly-Phe-NH_2_ and SHU9119).

Code	Compound	Affinity			Selectivity		
		MOR	DOR	MC4R	MOR/ DOR	MC4R/ MOR	MC4R/ DOR
		IC_50_ ± SD nM #	IC_50_ ± SD nM #	IC_50_ ± SD nM *			
1	ENK-d-Ala-SHU	69.58 ± 18.68	14.46 ± 1.91	0.07 ± 0.01	0.2	994	207
2	ENK-β-Ala-SHU	103.61 ± 29.10	22.80 ± 7.49	0.12 ± 0.01	0.2	863	190
3	ENK-Ahx-SHU	64.03 ± 34.64	45.43 ± 32.33	1.83 ± 0.81	0.7	35	25
4	ENK-(Ahx)_2_-SHU	5.47 ± 1.38	22.32 ± 12.67	0.50 ± 0.16	4.1	11	45
Tyr-d-Ala-Gly-Phe-NH_2_		12.77 ± 3.21	171.47 ± 114.61	-	13.4	-	-
SHU9119		----	---	0.15 ± 0.01			

# IC_50_ ± SD, half-maximal inhibitory concentration ± standard deviation of three determinations in duplicate, * IC_50_ values were calculated as an average of two separate competition assays performed in duplicates. In the calculation of **1**, one data point was removed due to being significant outlier.

## Data Availability

Not applicable.

## Supplementary Materials

Supplementary materials can be found at https://www.mdpi.com/article/10.3390/ijms23020674/s1.
