# Supplementary material for: Bifunctional Opioid/Melanocortin Peptidomimetics for Use in Neuropathic Pain: Variation in the Type and Length of the Linker Connecting the Two Pharmacophores
Source: Int J Mol Sci. 2022 Jan 8;23(2):674. doi: 10.3390/ijms23020674 (PMC8775902; doi:10.3390/ijms23020674)
Supplement: Supplementary file 1 [file ijms-23-00674-s001.zip › ijms-1496746-supplementary.pdf]

Supplementary Materials to

Bifunctional opioid / melanocortin peptidomimetics for use in neuropathic pain: variation in the type and length of the linker connecting the two pharmacophores

Ewa Witkowska [1,*], Magda Godlewska [1], Jowita Osiejuk [1], Sandra Gątarz [1], Beata Wileńska [1,2], Katarzyna Kosińska [1], Joanna Starnowska-Sokół [3], Anna Piotrowska [3], Piotr F. J. Lipiński [4], Joanna Matalińska [4], Jolanta Dyniewicz [4], Paweł K. Halik [5], Ewa Gniazdowska [5], Barbara Przewlocka [3] and Aleksandra Misicka [1,2,*]

1 Faculty of Chemistry, University of Warsaw, Pasteura 1, 02-093 Warsaw, Poland; magda.god@onet.pl (M.G.); jowita.osiejuk@student.uw.edu.pl (J.O.); sandra.gatarz@student.uw.edu.pl (S.G.); bwilenska@chem.uw.edu.pl (B.W.); katarzyna.kosinska2@student.uw.edu.pl (K.K.)

2 Biological and Chemical Research Centre, University of Warsaw, 101 Zwirki i Wigury St., 02-097 Warsaw, Poland

3 Department of Pain Pharmacology, Maj Institute of Pharmacology, Polish Academy of Sciences, 12 Smetna Str., 31-343 Krakow, Poland; joanna.starnowska@gmail.com (J.S.-S.); anna.piotrowskamurzyn@gmail.com (A.P.); barbara.przewlocka@gmail.com (B.P.)

4 Department of Neuropeptides, Mossakowski Medical Research Institute Polish Academy of Sciences, Pawińskiego 5, 02-106 Warsaw, Poland; plipinski@imdik.pan.pl (P.F.J.L.); jmatalinska@imdik.pan.pl (J.M.); jdyniewicz@imdik.pan.pl (J.D.)

5 Centre of Radiochemistry and Nuclear Chemistry, Institute of Nuclear Chemistry and Technology, Dorodna 16, 03-195 Warsaw, Poland; p.halik@ichtj.waw.pl (P.K.H.); e.gniazdowska@ichtj.waw.pl (E.G.)

* Correspondence: ewawit@chem.uw.edu.pl (E.W.); misicka@chem.uw.edu.pl (A.M.)

Table of contents

Figure S1. General scheme for the synthesis of peptidomimetics **1-8** ……………………………....……......SI-2
Analytical and spectroscopic methods ……………………………………………………….…………SI-3
Table S1. Analytical data of synthesized peptidomimetics **1-9** ……………………………………...SI-3
Chromatograms and mass spectra of purified compounds 1-9 ……………………………………SI-4
Figure S2. Chromatogram and mass spectra of purified compound **1** ……………………...…………..SI-4
Figure S3. Chromatogram and mass spectra of purified compound **2** …………………………………..…..SI-4
Figure S4. Chromatogram and mass spectra of purified compound **3** …………………………………..…..SI-4
Figure S5. Chromatogram and mass spectra of purified compound **4** ……………………………....……....SI-5
Figure S6. Chromatogram and mass spectra of purified compound **5** …………………………………..…..SI-5
Figure S7. Chromatogram and mass spectra of purified compound **6** …………………………………..…..SI-5
Figure S8. Chromatogram and mass spectra of purified compound **7** …………………………………..…..SI-6
Figure S9. Chromatogram and mass spectra of purified compound **8** …………………………………..…..SI-6
Figure S10. Chromatogram and mass spectra of purified compound **9** ……………………………........SI-6
Table S2. Lipophilicity of compounds **1-4** ……………………………………………………...……....SI-7
Docking of compounds 1-4 to MOR.
Table S3. Energetics of the top scored clusters (at MOR). . ……………..……………………….…....…SI-8
Figure S11. Binding poses of compounds **1-4** and DAMGO in the MOR binding site. Focus on the opioid fragment. ……………………………………………………………………………………………..…SI-8
Figure S12. Binding poses of compounds **1-4** in the MOR binding site. Focus on the melanocortin-pharmacophoric fragment………………………………………………………………………….…SI-9
Contacts of the melanocortin-pharmacophoric fragment of compounds 1-4 with MOR ………..….SI-9
Docking of compounds 1-4 to DOR.
Table S4. Energetics of the top scored clusters (at DOR). . ……………..…………………………..…..SI-10
Figure S13. Binding poses of compounds **1-4** in the DOR binding site. Focus on the opioid fragment. .SI-10
Figure S14. Binding poses of compounds **1-4** in the DOR binding site. Focus on the melanocortin-pharmacophoric fragment. . ……………..…………………………………………………..……..….…SI-11
Docking of compounds 1-4 to MC4R.
Table S5. Energetics of the top scored clusters (at MC4R). …..……………..…………………….…SI-12
Listing of interactions of the opioid-linker fragment found in complexes of 3 with MC4R …..…...SI-12
Figure S15. Binding poses of compounds **1-4** and of SHU9119 at the MC4R receptor. ………………SI-12
Figure S16. Binding poses of compounds **1-4** at the MC4R receptor (side-view). Focus on the positioning

of the opioid-linker fragment. ……………………………………...…………………………………..SI-13

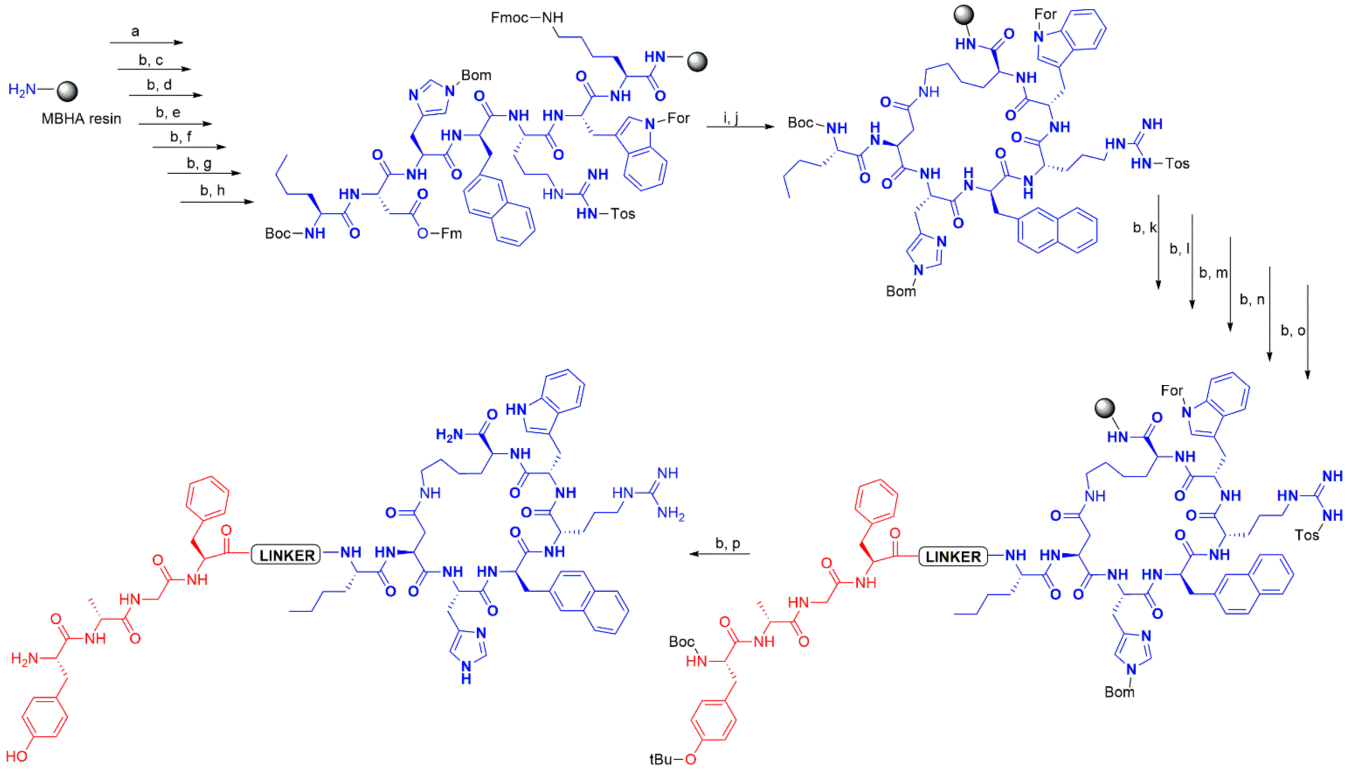

Figure S1. General scheme for the synthesis of peptidomimetics **1**-**8**: a) Boc-Lys(Fmoc)-OH, DIC, HOBt; b) 50% TFA in DCM; c) Boc-Trp(For)-OH, DIC, HOBt; d) Boc-Arg(Tos)-OH, DIC, HOBt; e) Boc-D-Nal(2')-OH, DIC, HOBt; f) Boc-His(Bom)-OH, DIC, HOBt; g) Boc-Asp(OFm)-OH, DIC, HOBt; h) Boc-Nle-OH, DIC, HOBt; i) 30% piperidine in DMF; j) DIC, HOBt; k) protected linker (Boc-D-Ala-OH or Boc-β-Ala-OH or Fmoc-Ahx-OH or Fmoc-4-AMB-OH or Fmoc-4-APhAc-OH or Boc-Gly-OH, Boc-Pro-OH), DIC, HOBt; l) Boc-Phe-OH, DIC, HOBt; m) Boc-Gly-OH, DIC, HOBt; n) Boc-D-Ala-OH, DIC; HOBt; o) Boc-Tyr(tBu)-OH, DIC, HOBt; p) HF

In the case of compound **9**, instead of tyrosine in the first position, 2,6-dimethyltyrosine (Dmt) is used.

Analytical and spectroscopic methods

Purification and analysis of synthetized compounds **1-**9 were performed on a Knauer HPLC system. The peptidomimetics were purified by semipreparative RP HPLC with column : Neukleosil 300 C18 (5 µm, 8 x 250 mm). A solvent system was used - A: 0,1% TFA in water, B: 80% MeCN in A, with flow rate 2.0 mL/min. The fractions were analyzed with the same system using a Eurospher 100 C18 reversed-phase analytical column (5 µm, 4.6 x 250 mm) and the same solvent systems (1.0 mL/min, t= 20 min). Detection was at 220 nm. Mass spectra were registered on the Shimadzu LC-MS mass spectrometer provided with an ESI ion source (electrospray), ion trap and time-of-flight analyzer Analyses were carried out in the positive ion mode. For compound **9** MALDI Shimadzu Biotech Axima Performance with time-of-flight analyzer and the cinnamic acid as the matrix was used.

Table S1. Analytical data of synthesized peptidomimetics **1-9**

**Code**	**Compound**	**Molecular formula**	**Molecular weight (g/mol)**	**ESI MS Ion $[M + 3H]^{3+}$**		**HPLC[a] (t_R, min)**
				**m/z calcd**	**m/z obsd**	
**1**	ENK-D-Ala-SHU	$C_{78}H_{100}N_{20}O_{14}$	1540.8	514.6	514.6	13.2[b]
**2**	ENK-β-Ala-SHU	$C_{78}H_{100}N_{20}O_{14}$	1540.8	514.6	514.6	12.1[b]
**3**	ENK-Ahx-SHU	$C_{81}H_{106}N_{20}O_{14}$	1583.8	528.6	528.9	13.2[b]
**4**	ENK-(Ahx)$_2$-SHU	$C_{87}H_{117}N_{21}O_{15}$	1697.0	566.6	566.6	12.3[b]
**5**	ENK-4AMB-SHU	$C_{83}H_{102}N_{20}O_{14}$	1603.8	535.6	535.6	13.2[c]
**6**	ENK-4APhAc-SHU	$C_{83}H_{102}N_{20}O_{14}$	1603.8	535.6	535.3	14.3[b]
**7**	ENK-Pro-Gly-SHU	$C_{82}H_{105}N_{21}O_{15}$	1624.8	542.6	542.3	10.7[d]
**8**	ENK-(Pro-Gly)$_2$-SHU	$C_{89}H_{115}N_{23}O_{17}$	1778.9	594.0	593.6	10.3[d]
**9**	[Dmt[1]]ENK-Ahx-SHU[e]	$C_{83}H_{110}N_{20}O_{14}$	1611.8[f]			10.9[d]

[a] Peformed on a Knauer system (Eurospher 100- C18, 4.6 mm x 250 mm, 5 µm), time of analysis 20 min, v=1ml/min; [b,c,d] HPLC solvent system A 0.1% TFA in water, B 80% MeCN in A, [b] programme 40-70% B, [c] programme 30-70% B, [d] programme 40-80% B; [e] Dmt – 2,6-dimethyltyrosine; [f] MALDI MS analysis of 9: calcd 1611.8 obsd 1611.4;

Chromatograms and mass spectra of purified compounds 1-9

1 ENK-D-Ala-SHU (Tyr-D-Ala-Gly-Phe-D-Ala-Nle-c[Asp-His-D-Nal(2')-Arg-Trp-Lys]-NH$_2$) $C_{78}H_{100}N_{20}O_{14}$ MW 1540.8 g/mol

A

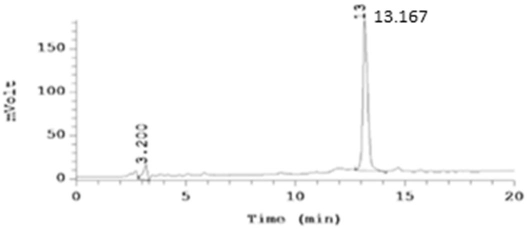

B

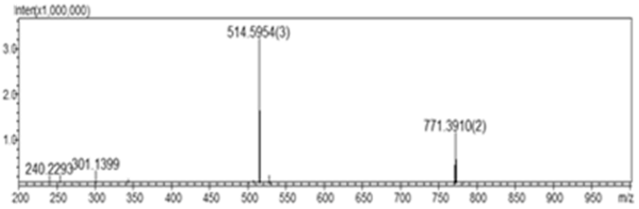

Figure S2. Chromatogram and mass spectra of purified compound **1** A) RP HPLC chromatogram of **1**, linear gradient 40-70% B, t_R 13.2 min; B) ESI MS of **1** m/z $[M+2H]^{2+}$ calcd 771.4, obsd 771,4, $[M+3H]^{3+}$ calcd 514.6, obsd 514.6.

2 ENK-β-Ala-SHU (Tyr-D-Ala-Gly-Phe-β-Ala-Nle-c[Asp-His-D-Nal(2')-Arg-Trp-Lys]-NH$_2$) $C_{78}H_{100}N_{20}O_{14}$ MW 1540.8 g/mol

A

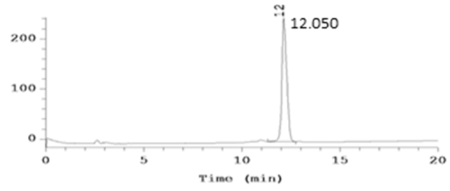

B

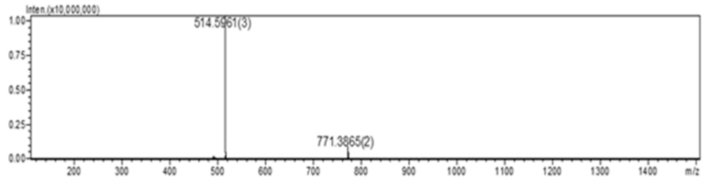

Figure S3. Chromatogram and mass spectra of purified compound **2** A) RP HPLC chromatogram of **2**, linear gradient 40-70% B, t_R 12.1 min; B) ESI MS of **2** m/z $[M+2H]^{2+}$ calcd 771.4, obsd 771,4, $[M+3H]^{3+}$ calcd 514.6, obsd 514.6.

3 ENK-Ahx-SHU (Tyr-D-Ala-Gly-Phe-Ahx-Nle-c[Asp-His-D-Nal(2')-Arg-Trp-Lys]-NH$_2$) $C_{81}H_{106}N_{20}O_{14}$ MW 1583.8 g/mol

A

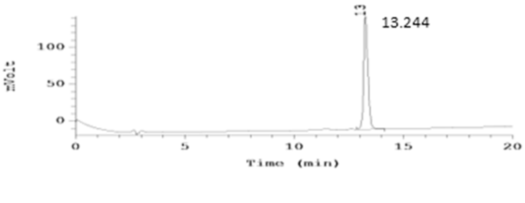

B

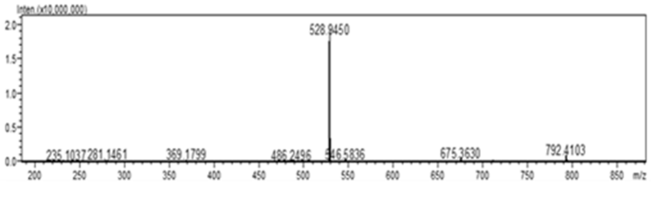

Figure S4. Chromatogram and mass spectra of purified compound **3** A) RP HPLC chromatogram of **3**, linear gradient 40-70% B, t_R 13.2 min; B) ESI MS of **3** m/z $[M+3H]^{3+}$ calcd 528.6, obsd 528.9

4 ENK-(Ahx)$_2$-SHU (Tyr-D-Ala-Gly-Phe-Ahx-Ahx-Nle-c[Asp-His-D-Nal(2')-Arg-Trp-Lys]-NH$_2$)
$C_{87}H_{117}N_{21}O_{15}$ MW 1697.0 g/mol

A

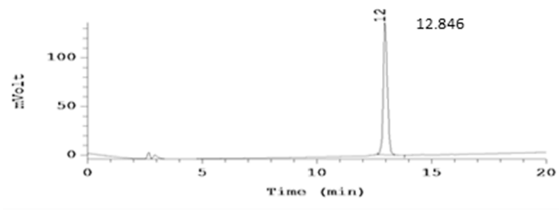

B

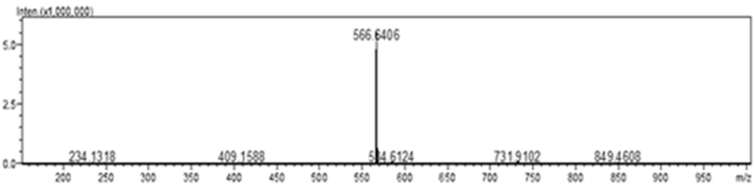

Figure S5. Chromatogram and mass spectra of purified compound **4** A) RP HPLC chromatogram of **4**, linear gradient 40-70% B, t_R 12.8 min; B) ESI MS of **4** m/z $[M+3H]^{3+}$ calcd 566.6, obsd 566.6

5 ENK-4AMB-SHU (Tyr-D-Ala-Gly-Phe-4AMB-Nle-c[Asp-His-D-Nal(2')-Arg-Trp-Lys]-NH$_2$)
$C_{83}H_{102}N_{20}O_{14}$ MW 1603.8 g/mol

A

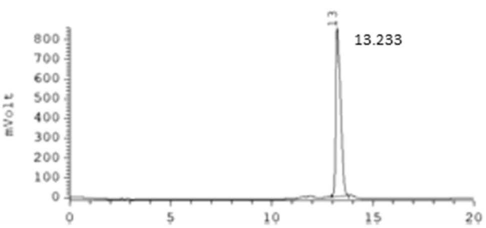

B

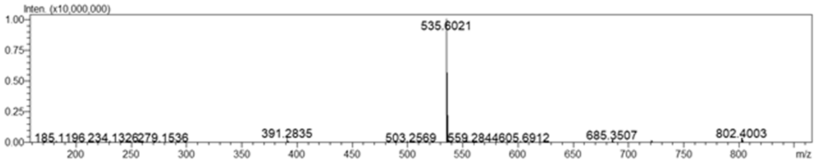

Figure S6. Chromatogram and mass spectra of purified compound **5** A) RP HPLC chromatogram of **5**, linear gradient 30-70% B, t_R 13.2 min; B) ESI MS of **5** m/z $[M+3H]^{3+}$ calcd, 535.6, obsd 535.6

6 ENK-4APhAc-SHU (Tyr-D-Ala-Gly-Phe-4APhAc-Nle-c[Asp-His-D-Nal(2')-Arg-Trp-Lys]-NH$_2$)
$C_{83}H_{102}N_{20}O_{14}$ MW 1603.8g/mol

A

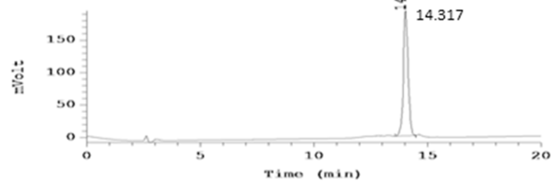

B

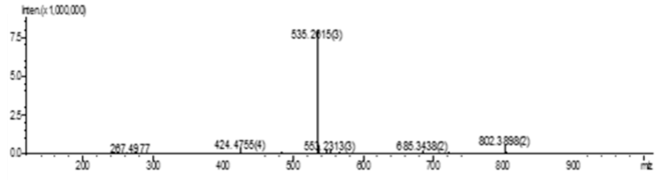

Figure S7. Chromatogram and mass spectra of purified compound **6** A) RP HPLC chromatogram of **6**, linear gradient 40-70% B, t_R 14.3 min; B) ESI MS of **6** m/z $[M+3H]^{3+}$ calcd, 535.6, obsd 535.3

7 ENK-Pro-Gly-SHU (Tyr-D-Ala-Gly-Phe-Pro-Gly-Nle-c[Asp-His-D-Nal(2')-Arg-Trp-Lys]-NH_2)
$C_{82}H_{105}N_{21}O_{15}$ MW 1624.8 g/mol

A

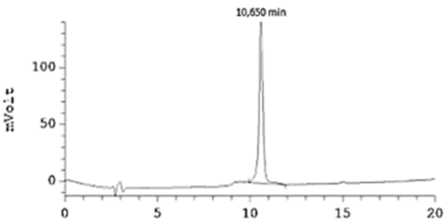

B

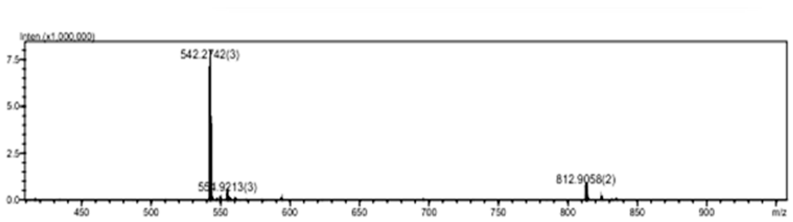

Figure S8. Chromatogram and mass spectra of purified compound **7** A) RP HPLC chromatogram of **7**, linear gradient 40-80% B, t_R 10.7 min; B) ESI MS of **7** m/z $[M+3H]^{3+}$ calcd 542.6, obsd 542.3

8 ENK-(Pro-Gly)$_2$-SHU (Tyr-D-Ala-Gly-Phe-(Pro-Gly)$_2$-Nle-c[Asp-His-D-Nal(2')-Arg-Trp-Lys]-NH_2)
$C_{89}H_{115}N_{23}O_{17}$ MW 1778.9 g/mol

A

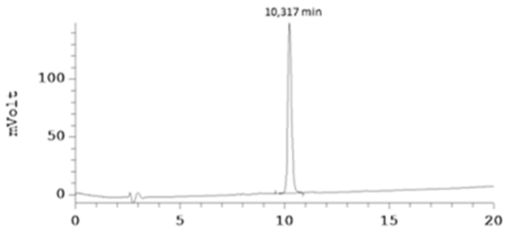

B

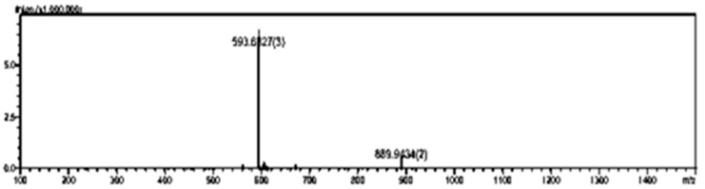

Figure S9. Chromatogram and mass spectra of purified compound **8** A) RP HPLC chromatogram of **8**, linear gradient 40-80% B, t_R 10.3 min; B) ESI MS of **8** m/z $[M+3H]^{3+}$ calcd 594.0, obsd 593.6

9 [Dmt[1]]ENK-Ahx-SHU (Dmt-D-Ala-Gly-Phe-Ahx-Nle-c[Asp-His-D-Nal(2')-Arg-Trp-Lys]-NH_2)
$C_{83}H_{110}N_{20}O_{14}$ MW 1611.8g/mol

A

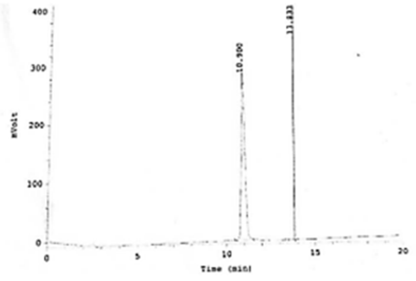

B

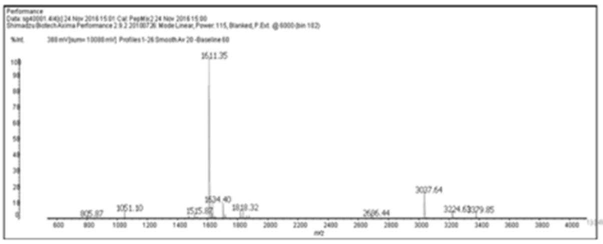

Figure S10. Chromatogram and mass spectra of purified compound **9** A) RP HPLC chromatogram of **9**, linear gradient 40-80% B, t_R 10.9 min; B) MALDI MS of **9** calcd 1611.8, obsd 1611.4

Table S2. Lipophilicity of compounds **1**-**4**.

Code	Compound	Lipophilicity	
		P	Log P
**1**	ENK-Ala-SHU	$(5.01 \pm 0.26) \times 10^{-3}$	-2.30 ± 0.02
**2**	ENK-β-Ala-SHU	$(4.66 \pm 0.22) \times 10^{-3}$	-2.33 ± 0.02
**3**	ENK-Ahx-SHU	$(3.75 \pm 0.12) \times 10^{-3}$	-2.43 ± 0.01
**4**	ENK-Ahx-Ahx-SHU	$(1.21 \pm 0.05) \times 10^{-3}$	-2.92 ± 0.02

Docking of compounds 1-4 to MOR.

Table S3. Energetics of the top scored clusters (at MOR).

		Lowest binding energy	Mean binding energy	Number in cluster
**Compounds**	**Cluster**	**[kcal/mol]**		
**1**	**A**	-8.11	-7.05	21
	**B**	-8.06	-7.18	299
**2**		-9.37	-8.40	56
**3**	**A**	-9.97	-8.59	11
	**B**	-9.96	-7.65	19
**4**		-9.56	-7.06	15

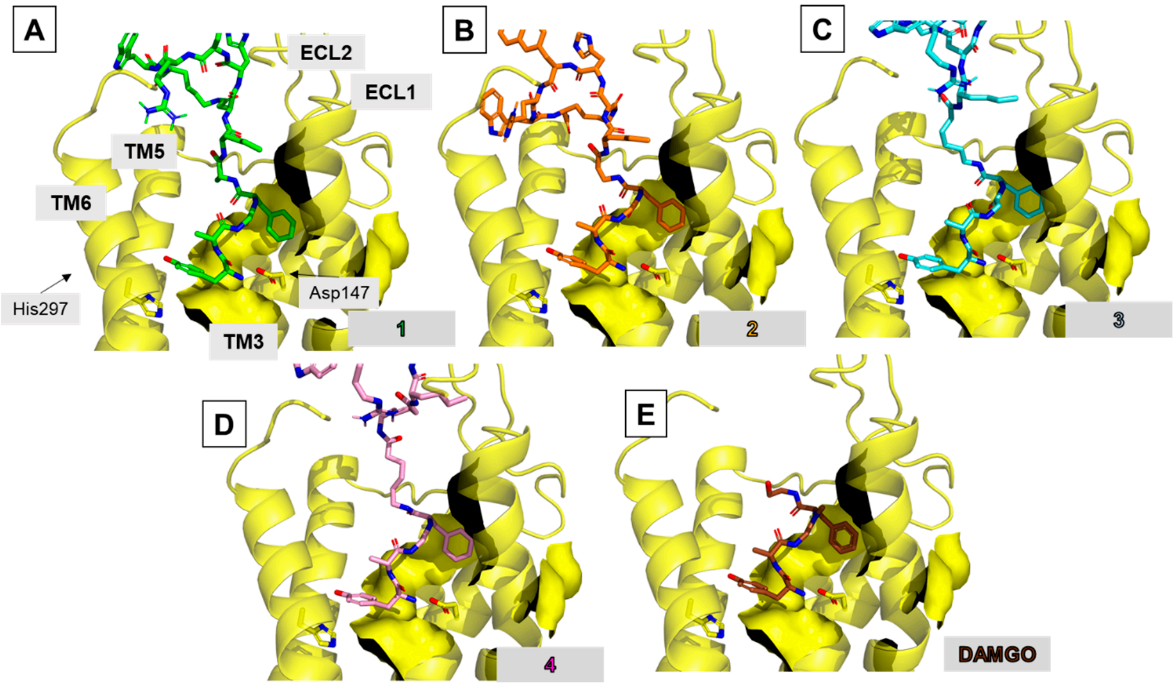

Figure S11. Binding poses of compounds **1-4** and DAMGO in the MOR binding site. Focus on the opioid fragment. The receptor (yellow) is shown in a simplified manner as helices (without TM1 and TM7), surface, and selected side chains shown as sticks. Ligands are shown as sticks. A) compound **1** (green), B) compound **2** (orange), C) compound **3** (aquamarine blue), D) compound **4** (pink), E) DAMGO (brown). For the sake of clarity only selected receptor elements and residues are labelled in part A. In the remaining parts, identical orientation is preserved.

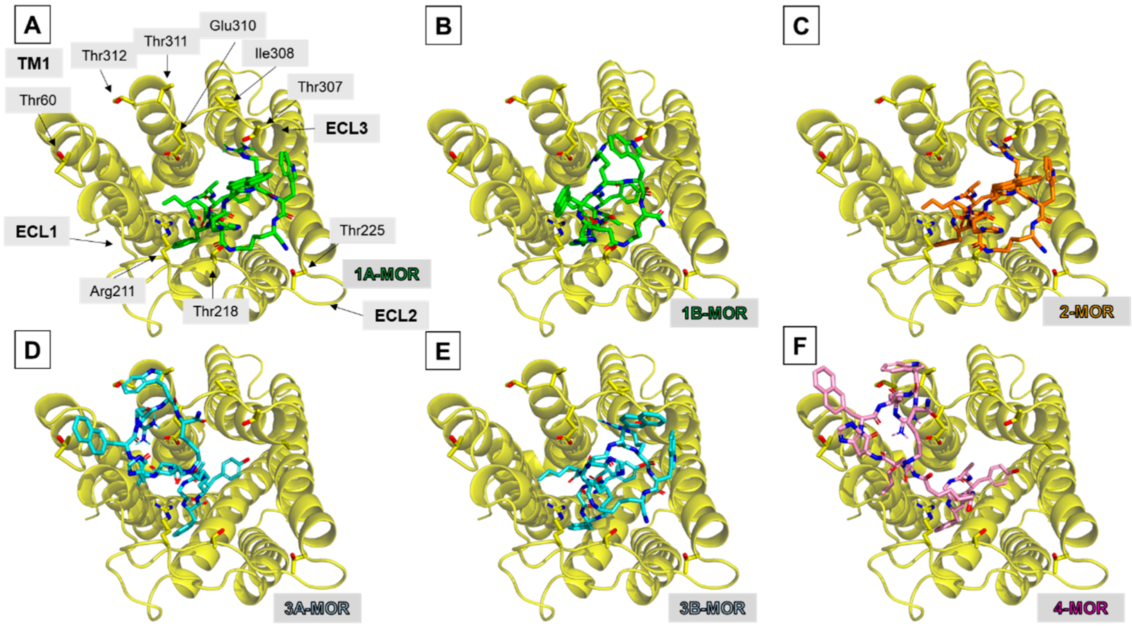

Figure S12. Binding poses of compounds **1-4** in the MOR binding site. Focus on the melanocortin-pharmacophoric fragment. The receptor (yellow) is shown in a simplified manner as helices and selected side chains shown as sticks. Ligands are shown as sticks. A) compound **1**, pose 1A-MOR (green), B) compound **1**, pose 1B-MOR (green), C) compound **2** (orange), D) compound **3,** pose 3A-MOR (aquamarine blue), E) compound **3,** pose 3B-MOR (aquamarine blue), F) compound **4** (pink). For the sake of clarity only selected receptor elements and residues are labelled in part A. In the remaining parts, identical orientation is preserved.

Contacts of the melanocortin-pharmacophoric fragment of compounds 1-4 with MOR

For compound **1**, docking finds two top-scored clusters of solutions (**1A-MOR** and **1B-MOR**). In both, the opioid part is positioned in virtually identical manner (Figure S11), as described in the main body of the manuscript. The melanocortin-pharmacophoric fragment is wedged between ECL2 and ECL3 of the receptor (Figure S12A and S12B). In **1A-MOR** pose (Figure S12A), the exocyclic amide's hydrogen H-bonds to Thr225^{ECL2}. The side-chains Arg^{M8} and Trp^{M9} are involved in interactions to Ala304$^{6.59}$ carbonyl oxygen. The lactam bond in the peptide interacts with Thr218^{ECL2} side-chain. In the **1B-MOR** pose (Figure S12B), Arg^{M8} side chain locates closely to Ile308^{ECL3} and Glu310^{ECL3} side-chains, Trp^{M9} is placed near Thr307^{ECL3}, while His^{M6} approaches Arg211^{ECL2}.

For compound **2**, the MOR-bound pose (in the melanocortin-pharmacophoric fragment, Figure S12C) is stabilized by H-bond of the exocyclic amide's hydrogen to Thr225^{ECL2} side chain. The lactam bond of the peptide is located close to Thr218^{ECL2}. The side-chains of Arg^{M8} and Trp^{M9} are in contact with Ala304$^{6.59}$ (side-chain and carbonyl's oxygen). Additionally, Arg^{M8} interacts with Thr307^{ECL3} and Ile308^{ECL3} (side-chain and carbonyl's oxygen).

In the case of compound **3** (Figures S12D and S12E), two energetically close solutions (**3A-MOR** and **3B-MOR**) are found, differing only with respect to the positioning of the melanocortin-pharmacophoric fragment. In **3A-MOR** pose (Figures S12D), this fragment locates close to ECL3 and the extracellular tip of TM1. In this mode, the D-Nal(2')^{M7} side chain approaches Thr60$^{N\text{-term}}$, while Arg^{M8} side chain interacts with Glu310^{ECL3} and Thr311$^{7.28}$. In the **3B-MOR** pose (Figures S12E), the melanocortin-pharmacophoric fragment is wedged between ECL2 and ECL3. Among the intramolecular contacts, one finds interaction of Arg^{M8} side-chain with the side-chains of Thr307^{ECL3} and Glu310^{ECL3}, as well as of Trp^{M9} with Ala304$^{6.59}$.

In the case of the longest analogue **4**, the melanocortin-pharmacophoric fragment is placed near the extracellular tip of TM1 and ECL3 (Figure S12F). His^{M6} approaches Thr60$^{N\text{-term}}$. The side-chain of Arg^{M8} contacts to side-chains of Glu310^{ECL3} and Thr312$^{7.29}$, while the Trp^{M9} interacts with Thr311$^{7.28}$.

Docking of compounds 1-4 to DOR.

Table S4. Energetics of the top scored clusters (at DOR).

	Lowest binding energy	Mean binding energy	Number in cluster
**Compounds**	**[kcal/mol]**		
**1**	-6.59	-6.03	11
**2**	-6.51	-5.87	12
**3**	-7.17	-5.85	16
**4**	-5.47	-3.28	36

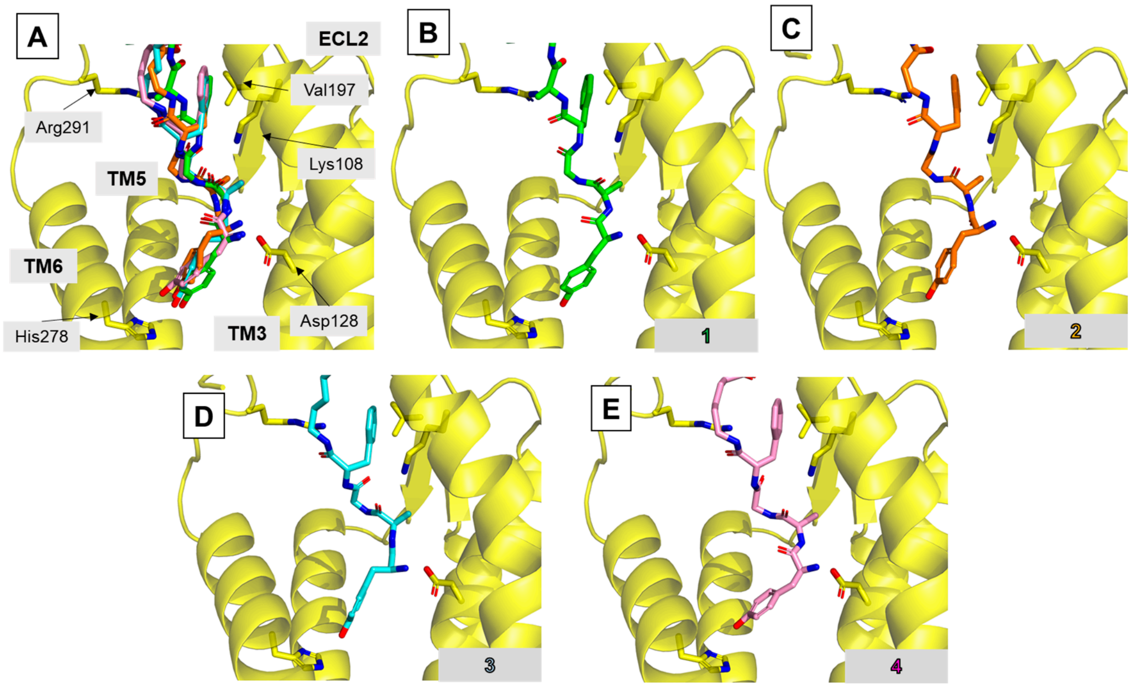

Figure S13. Binding poses of compounds **1**-**4** in the DOR binding site. Focus on the opioid fragment. The receptor (yellow) is shown in a simplified manner as helices (without TM1 and TM7), and selected side chains shown as sticks. Ligands are shown as sticks. A) Superposition of compounds **1**-**4**, B) compound **1** (green), C) compound **2** (orange), D) compound **3** (aquamarine blue), E) compound **4** (pink). For the sake of clarity only selected receptor elements and residues are labelled in part A. In the remaining parts, identical orientation is preserved.

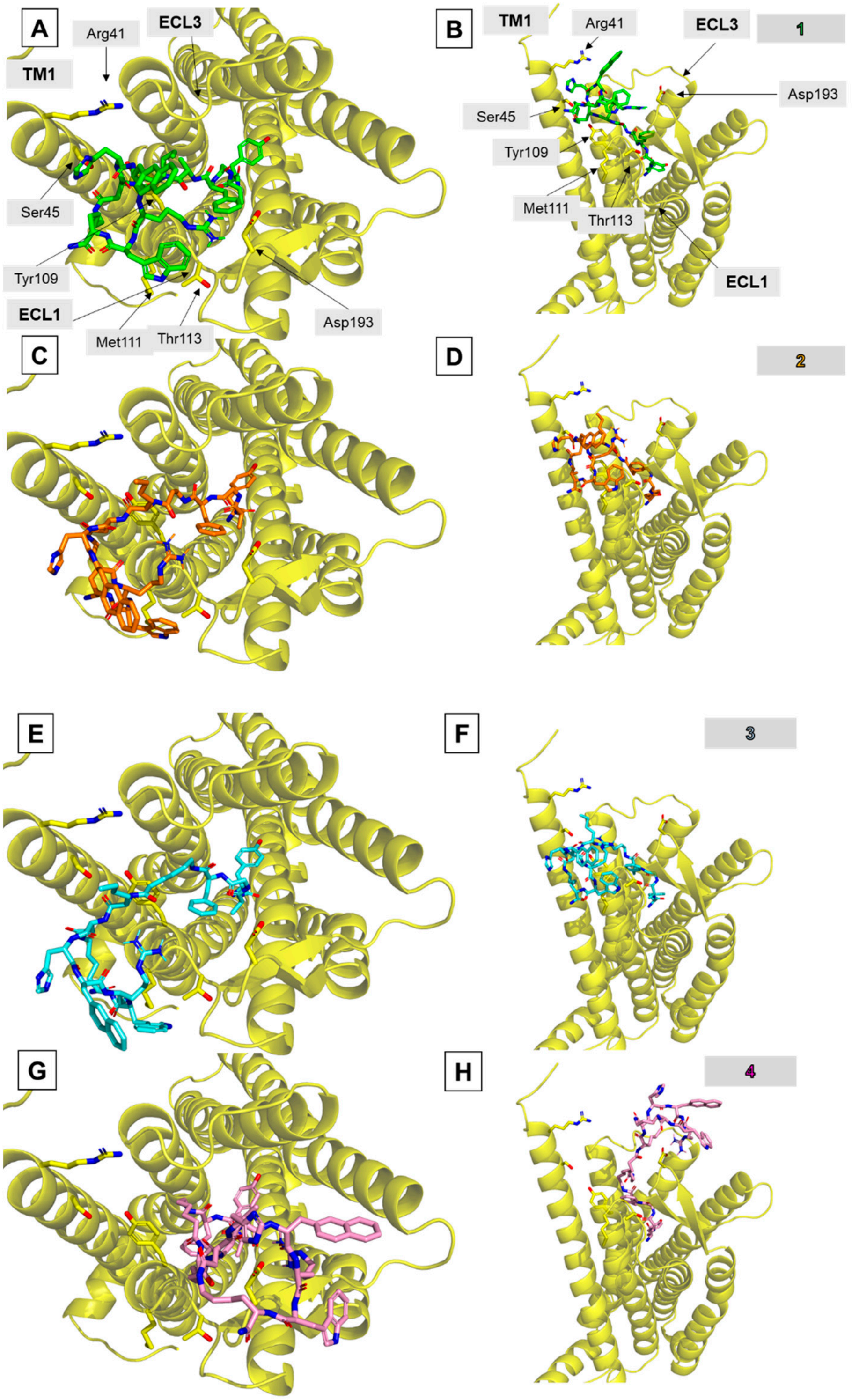

Figure S14. Binding poses of compounds **1**-**4** in the DOR binding site. Focus on the melanocortin-pharmacophoric fragment. The receptor (yellow) is shown in a simplified manner as helices and selected side chains shown as sticks. Ligands are shown as sticks. A-B) top and side view for compound **1** (green), C-D) top and side view for compound **2** (orange), E-F) top and side view for compound **3** (aquamarine blue), G-H) top and side view for compound **4** (pink). For the sake of clarity only selected receptor elements and residues are labelled in parts A and B. In the remaining parts, identical orientation is preserved.

Docking of compounds 1-4 to MC4R.

Table S5. Energetics of the top scored clusters (at MC4R).

		Lowest binding energy	Mean binding energy	Number in cluster
**Compounds**	**Cluster**	**[kcal/mol]**		
**1**		-20.32	-19.03	115
**2**		-19.26	-18.19	15
**3**	**A**	-19.62	-17.00	7
	**B**	-19.08	-17.27	13
	**C**	-19.04	-17.84	8
**4**		-19.76	-18.43	9

Listing of interactions of the opioid-linker fragment found in complexes of 3 with MC4R (three poses):

3A-MC4R

Tyr^{O1} NH3+ ⋯ Asp189^{ECL2} side chain (H-bond/ionic)

Nle^{M4} NH (amide) ⋯ Asp111^{ECL1} side chain (H-bond)

3B-MC4R

Tyr^{O1} NH3+ ⋯ Thr112^{ECL1} side chain (H-bond)

Phe^{O4} (aromatic ring) ⋯ Thr112^{ECL1} side chain (hydrophobic)

Nle^{M4} NH (amide) ⋯ Asp11 ^{ECL1} side chain (H-bond)

3C-MC4R

Gly^{O3} NH (amide) ⋯ Ile186$^{4.62}$ carbonyl oxygen (H-bond)

Phe^{O4} NH (amide) ⋯ Ile185$^{4.61}$ carbonyl oxygen (H-bond)

Phe^{O4} (aromatic ring) ⋯ Ile186$^{4.62}$ side chain (hydrophobic)

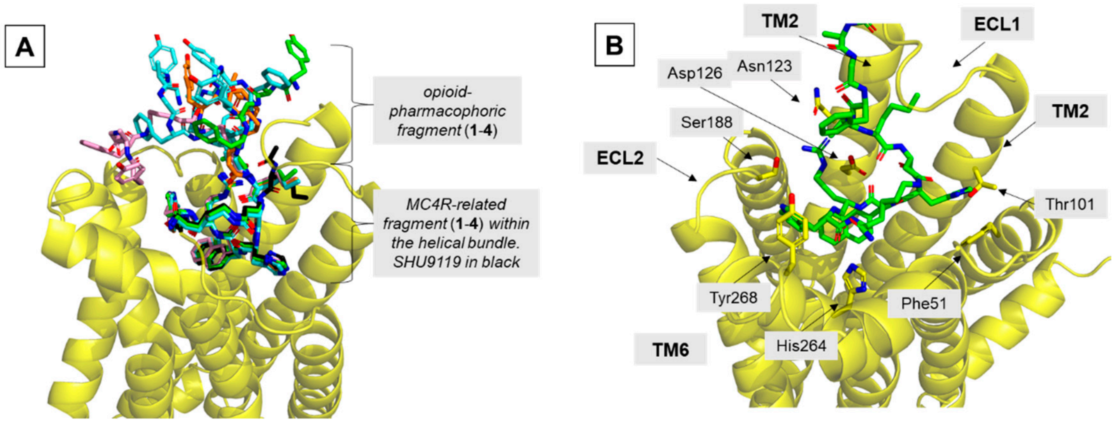

Figure S15. Binding poses of compounds **1**-**4** and of SHU9119 at the MC4R receptor. A) A general view, B) focus (rotated) on the interactions of the melanocortin-pharmacophoric fragment, compound **1** (green). Ligands are shown as sticks. SHU9119 is represented in black. The receptor (yellow) is shown in a simplified manner as helices and some side chains shown as sticks. Only selected residues of the receptor are labelled.

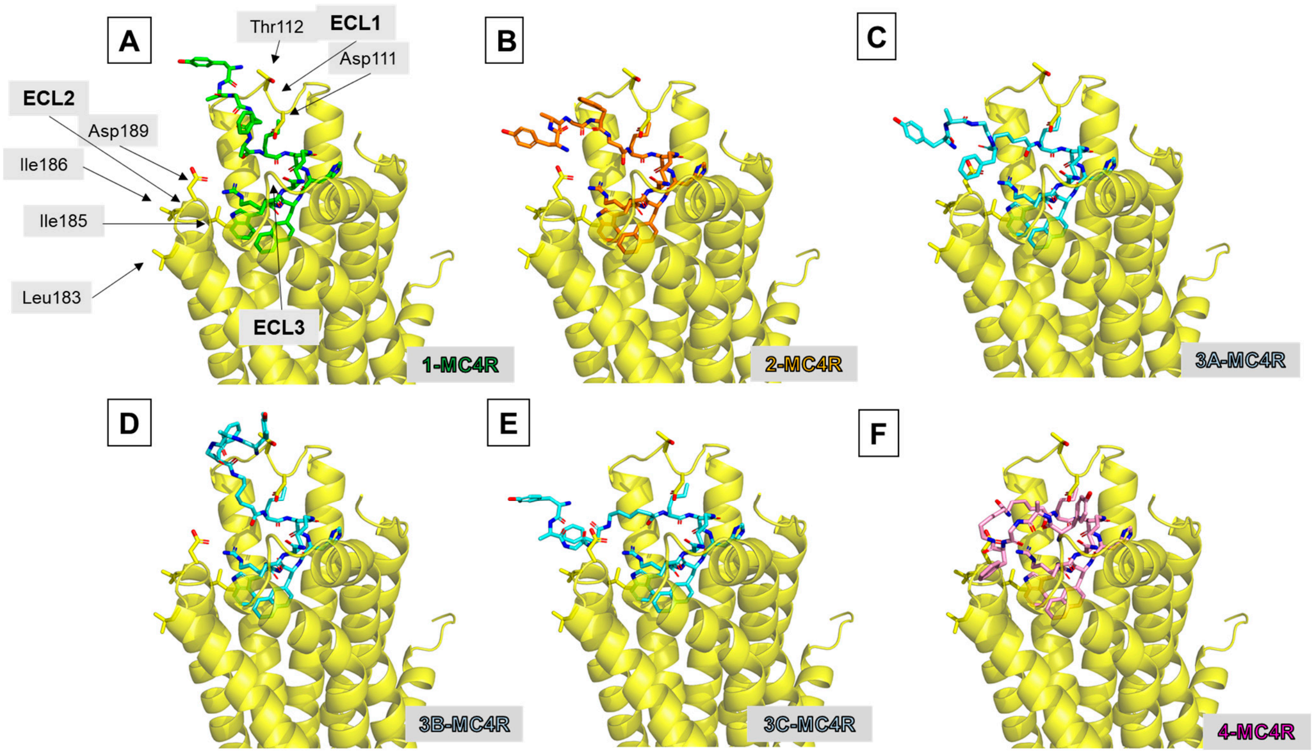

Figure S16. Binding poses of compounds **1**-**4** at the MC4R receptor (side-view). Focus on the positioning of the opioid-linker fragment. Ligands are shown as sticks. The receptor (yellow) is shown in a simplified manner as helices and some side chains shown as sticks. A) Compound **1** (green), B) compound **2** (orange), C) compound **3**, pose 3A-MC4R (aquamarine blue), D) compound **3**, pose 3B-MC4R (aquamarine blue), E) compound **3**, pose 3C-MC4R (aquamarine blue), F) compound **4** (pink). For the sake of clarity only selected receptor elements and residues are labelled in part. In the remaining parts, identical orientation is preserved.
